# Long‐Term Outcomes After Stoma Creation in Crohn's Disease: A Retrospective Cohort Study of 422 Patients

**DOI:** 10.1002/ags3.70219

**Published:** 2026-04-06

**Authors:** Kentaro Nagano, Ryuichi Kuwahara, Yusuke Tomoo, Kurando Kusunoki, Yuki Horio, Kei Kimura, Kozo Kataoka, Masataka Ikeda, Motoi Uchino, Hiroki Ikeuchi

**Affiliations:** ^1^ Division of Inflammatory Bowel Disease Surgery, Department of Gastroenterological Surgery Hyogo Medical University Nishinomiya Japan; ^2^ Division of Lower Gastroenterological Surgery, Department of Gastroenterological Surgery Hyogo Medical University Nishinomiya Japan

**Keywords:** anorectal disease, biologic therapy, Crohn's disease, long‐term outcomes, stoma

## Abstract

**Aim:**

Stoma creation is often required in Crohn's disease (CD), most commonly because of progressive anorectal disease. As stoma closure strongly influences long‐term quality of life and surgical decision‐making, this study evaluated the indications and long‐term outcomes of stoma creation in patients with CD.

**Methods:**

A prospectively maintained database of CD patients who underwent surgery at Hyogo Medical University between 1974 and 2017 was retrospectively reviewed. Among 1440 surgically treated patients, 422 adults who required stoma creation for CD‐related indications were included. A permanent stoma was defined as resulting from abdominoperineal resection or remaining unclosed for more than 24 months. Permanent stoma‐free survival was estimated using the Kaplan–Meier method.

**Results:**

Of the 422 patients, 107 (25%) had a permanent stoma at initial creation, whereas 315 (75%) received a temporary stoma. Among temporary stomas, 98 (31%) were closed, 133 (42%) remained unclosed, and 84 (27%) became permanent; 13 patients (13%) required re‐stoma creation after stoma closure. Anorectal disease was the most common indication (224 patients, 71%), followed by poor bowel condition (75 patients, 24%) and anastomotic leakage (14 patients, 4%). Closure rates were 13% for anorectal disease and 56% for poor bowel condition. Permanent stoma‐free survival was 94.8% at 10 years and 82.7% at 20 years; in patients treated after 2005, these rates improved to 95.9% and 86.5%, respectively (*p* = 0.032).

**Conclusion:**

Among stomas created for Crohn's disease–related anorectal lesions, only 13% were successfully closed. These findings highlight the importance of achieving adequate disease control, including anorectal involvement, to avoid stoma creation.

## 1 Introduction

Inflammatory bowel disease (IBD), which mainly includes ulcerative colitis (UC) and Crohn's disease (CD), features a complex etiology involving environmental factors, genetic susceptibility, and alterations in the intestinal microbiota. Approximately 4.9 million individuals are affected by IBD worldwide, and the prevalence of both UC and CD continues to increase, particularly in newly industrialized countries [1, 2]. CD is characterized by chronic inflammation, and although any part of the gastrointestinal tract may be involved, anorectal lesions are frequently observed. In Western countries, the prevalence of anorectal involvement ranges from 4% to 13.7% [3, 4, 5], whereas studies from Asia have reported much higher rates of 28.8% to 39%. A recent prospective cohort study from Japan in 2022 demonstrated an especially high prevalence of 48.9% [6, 7, 8]. During the clinical course of CD, stoma creation is sometimes needed because of worsening anorectal disease, anorectal cancer, and impaired bowel function caused by complications such as perforation or postoperative anastomotic leakage. The cumulative incidence of stoma creation is reported to be 1.3% at 1 year, 1.9% at 3 years, and 2.5% at 5 years [9]. Temporary stomas are most commonly required due to the exacerbation of anorectal disease, whereas some patients require abdominoperineal resection and permanent stoma creation because of anorectal cancer [10, 11, 12].

However, no studies have examined the clinical course of stoma creation stratified by its underlying indication, and long‐term outcomes in this population remain unclear. Therefore, this study aimed to clarify the clinical characteristics, indications for stoma creation, and long‐term outcomes of CD patients who underwent stoma creation at our institution.

## 2 Materials and Methods

### 2.1 Patients and Data Collection

Between September 1974 and December 2017, 1440 patients with CD underwent surgery at our institution. Among these, 422 adult patients who required at least one stoma creation for CD‐related indications were included in the analysis. Patients younger than 18 years at the time of their initial stoma creation were excluded.

A prospectively maintained institutional database was used in this study. The database contains demographic information, clinical findings, operative details, and postoperative outcomes and is updated whenever a patient undergoes stoma creation. These data were retrospectively reviewed for the present analysis.

### 2.2 Surgical Management

Indications for surgery were discussed among IBD specialists, and the final decision was made jointly with the patient. All procedures were performed by colorectal surgeons experienced in the surgical management of CD. Postoperative medical therapy, including immunomodulators and biologic agents, was initiated 2–4 weeks after surgery according to the judgment of the treating gastroenterologist.

### 2.3 Definitions

A permanent stoma was defined as [1] a stoma resulting from abdominoperineal resection or [2] a stoma that remained unclosed for more than 24 months after creation. Poor conditions of the intestinal tract included severe malnutrition, complicated intra‐abdominal abscess or perforation with severe intra‐abdominal contamination, and other cases in which safe anastomosis could not be performed because of marked intestinal edema at the planned anastomosis site. Anorectal disease was defined as one or more CD–related anorectal manifestations, including perianal fistula, anorectal abscess, and anorectal stricture/stenosis; multiple lesions could coexist in the same patient. Preoperative medical therapy was defined as treatment with relevant agents within 3 months before surgery.

### 2.4 Statistical Analysis

Stoma closure rates and cumulative permanent stoma rates were estimated using the Kaplan–Meier method, with the time origin defined as the time of CD diagnosis.

Although infliximab was approved for Crohn's disease in Japan in 2002, its widespread use in routine clinical practice occurred several years later. Therefore, we selected 2005 as the cutoff year to reflect real‐world treatment patterns in Japan, supported by nationwide real‐world data demonstrating a marked increase in biologic use after the mid‐2000s [13]. Comparisons were performed using the log‐rank test. Continuous variables are presented as median (range), whereas categorical variables are expressed as numbers and percentages. All analyzes were conducted using JMP version 19 (SAS Institute Inc., Cary, NC, USA).

### 2.5 Ethical Considerations

All study protocols were approved by the Institutional Review Board of Hyogo Medical University (No. 4678). Due to the retrospective design of the study, the requirement for written informed consent was waived, and information about the study was disclosed on the institutional website to provide patients with the opportunity to opt out.

## 3 Results

### 3.1 Patient Characteristics

Table 1 shows the baseline characteristics of the 422 patients included in this study.

**TABLE 1:** Patients background.

Variable	*n* = 422
Sex (Male)	227 (53.7%)
Montreal Classification	
Age at diagnosis (A1/A2/A3)	93/303/26
Location (L1/L2/L3)	44/77/301
Behavior (B1/B2/B3)	28/136/258
Perianal disease (p)	321
Age at CD diagnosis (years)	21 (7–68)
Age at first stoma creation (years)	38 (15–75)
Duration of disease (months)	174 (0–603)
Smoking history	97 (22.9%)
Alcohol consumption	52 (12.3%)

*Note:* Baseline characteristics of 422 patients with Crohn's disease in whom stoma creation was performed.

The cohort consisted of 227 men (53.7%) and 145 women. According to the Montreal classification, 44 patients were classified as L1, 77 as L2, and 301 as L3. Regarding disease behavior, 28 patients were classified as B1, 136 as B2, and 258 as B3. Anorectal lesions were present in 321 patients (76.1%). The median age at CD diagnosis was 21 years (range, 7–68), and the median age at first stoma creation was 38 years (range, 15–75). The median disease duration was 174 months (range, 0–603). A smoking history was reported in 97 patients (22.9%), and alcohol consumption was reported in 52 patients (12.3%). Smoking history and alcohol consumption were defined as current or former use.

### 3.2 Preoperative Medical Treatments

Table 2 shows the preoperative medical treatments administered within 3 months before surgery. Elemental diet therapy was used in 146 patients (34.6%). A total of 301 patients (71.3%) received 5‐aminosalicylic acid, and 146 patients (34.6%) received corticosteroids. With respect to biologic therapy, 103 patients (24.4%) received infliximab, 31 patients (7.3%) received adalimumab, and no patients received preoperative treatment with other advanced therapies.

**TABLE 2:** Pre‐operative medical treatment.

Treatment	*n* = 422
Elemental diet therapy	146 (34.6%)
5‐aminosalicylic acid	301 (71.3%)
Corticosteroids	146 (34.6%)
Infliximab	103 (24.4%)
Adalimumab	31 (7.3%)
Other advanced therapies	0 (0%)

*Note:* Preoperative medical treatments administered within 3 months before surgery.

### 3.3 Clinical Course After Stoma Creation

**FIGURE 1:**
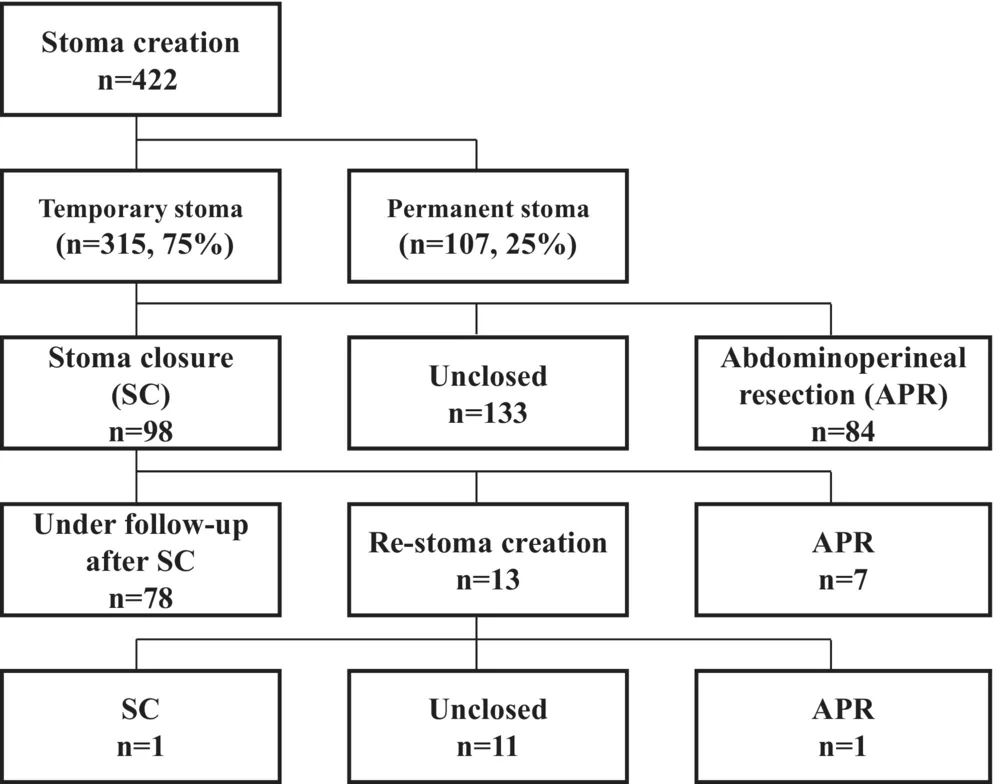
Clinical course of 422 patients with Crohn's disease in whom stoma creation was performed.

The clinical course of the 422 patients who underwent stoma creation is illustrated in Figure 1. At the initial procedure, 107 patients (25%) had a permanent stoma, whereas 315 patients (75%) received a temporary stoma. Regarding the anatomical origin of the stoma at the initial creation, among the 107 patients who had a permanent stoma at the initial surgery, 61 patients (57.0%) underwent small‐intestinal stoma creation and 46 patients (43.0%) underwent colonic stoma creation. Among the 315 patients who initially underwent temporary stoma creation, 271 patients (86.0%) had a small‐intestinal stoma and 44 patients (14.0%) had a colonic stoma. Among the 315 patients with temporary stomas, 84 (27%) eventually required abdominoperineal resection and permanent stoma creation, 133 (42%) had stomas that remained unclosed, and 98 (31%) achieved stoma closure.

Among the 98 patients who achieved closure, 7 patients (7%) subsequently required abdominoperineal resection with permanent stoma creation, 78 (80%) remained stoma‐free during follow‐up, and 13 (13%) required stoma recreation. Among the 13 patients who underwent stoma recreation, 1 patient (8%) ultimately required abdominoperineal resection, 11 patients (84%) had stomas that remained unclosed, and 1 patient (8%) achieved a second successful closure and continued follow‐up. Among the 422 patients, stoma‐related complications requiring reoperation were observed in 23 patients. These complications included stenosis adjacent to the stoma in 19 patients, fistula in 2, stoma prolapse in 1, and parastomal hernia in 1. Importantly, stenosis adjacent to the stoma was considered to be caused by Crohn's disease–related lesions rather than technical or mechanical stoma complications.

### 3.4 Factors That Led to Temporary Stoma Creation

Among the 315 patients who underwent temporary stoma creation, the most common indication was the progression of anorectal lesions, which accounted for 224 patients (71.1%). Poor intestinal conditions, which included severe edema of the bowel wall and the need to avoid anastomosis, were observed in 75 patients (23.8%). Anastomotic leakage led to stoma creation in 14 patients (4.4%), whereas other rare indications were identified in 2 patients (0.6%) (Table 3). The two cases categorized as “Other causes” involved patients who were initially diagnosed with ulcerative colitis and subsequently reclassified as Crohn's disease after surgery, with fistula formation originating from the ileal pouch.

**TABLE 3:** Factors in temporary stoma creation.

Factors	*n* = 315
Progression of anorectal lesions	224 (71.1%)
Poor condition of the intestinal tract	75 (23.8%)
Anastomotic leakage	14 (4.4%)
Other causes	2 (0.6%)

*Note:* Factors leading to temporary stoma creation in patients with Crohn's disease.

### 3.5 Cases That Required Abdominoperineal Resection or Stoma Recreation After Stoma Closure

Among the 98 patients who initially achieved stoma closure, 20 later required either abdominoperineal resection (APR) or restoma creation. APR was performed in seven cases, with six due to progression of anorectal lesions and 1 due to rectal cancer. Re‐stoma creation after stoma closure was required in 13 patients, primarily because of recurrent anorectal lesions (*n* = 11), followed by gastrointestinal perforation (*n* = 1) and rectal cancer (*n* = 1) (Table 4).

**TABLE 4:** Cases requiring abdominoperineal resection (APR) or re‐stoma creation after stoma closure.

Surgical technique	Factors	Number of cases
Abdominoperineal resection	Progression of anorectal lesions	6
	Rectal cancer	1
Re‐stoma creation	Progression of anorectal lesions	11
	Gastrointestinal perforation	1
	Rectal cancer	1

### 3.6 Final Outcomes According to the Indications for Temporary Stoma Creation

Among the 315 patients who underwent temporary stoma creation, the final outcomes included APR, permanent stoma without closure, or successful stoma closure. Overall, 92 patients (29.2%) had APR as the final outcome, whereas stoma closure was achieved in 98 patients (31.1%), including 79 patients (25.1%) who remained stoma‐free without re‐creation. Among patients who achieved stoma closure, 20 (20.4%) required subsequent re‐stoma creation, including eight patients (8.2%) who eventually underwent APR. The median follow‐up period after stoma closure was 13.1 years (range, 2.0–34.2 years). The long‐term outcomes of patients who underwent temporary stoma creation were evaluated according to the factors that led to stoma creation (Table 5). Among the 224 patients who required stoma creation because of the progression of anorectal lesions, 74 (33%) ultimately required APR, 108 (48%) remained unclosed, and 42 (19%) achieved stoma closure. After closure, 5 patients underwent APR, and 8 required stoma recreation. In contrast, among 75 patients who required stoma creation because of poor intestinal condition, 46 (61%) achieved closure, 22 (29%) had stomas that remained unclosed, and 7 (9%) ultimately required APR. After closure, two patients each underwent APR and stoma recreation. Among the 14 patients who developed anastomotic leakage, 8 (57%) successfully achieved stoma closure, 3 (21%) had stomas that remained unclosed, and 3 (21%) required APR. Both patients who required stoma creation for other causes achieved closure, although 1 required stoma recreation during follow‐up. Notably, 87% of patients who required stoma creation due to anorectal disease ultimately developed a permanent stoma. In contrast, patients who required stoma creation because of poor intestinal condition (*n* = 75) or anastomotic leakage (*n* = 14) had better outcomes, and of these, bowel continuity was maintained in 56% and 43% of patients, respectively, and thus permanent stoma creation over long‐term follow‐up was successfully avoided.

**TABLE 5:** Long‐term outcomes of patients who underwent temporary stoma creation, according to the factors leading to stoma creation.

	APR (*n* = 84)	Unclosed (*n* = 133)	Closed (*n* = 98)	APR after SC (*n* = 7)	Re‐stoma after SC (*n* = 13)	Under observation after SC (*n* = 78)
Progression of anorectal lesions (*n* = 224)	74	108	42	5	8	29 (13%)
Poor condition of the intestinal tract (*n* = 75)	7	22	46	2	2	42 (56%)
Anastomotic leakage (*n* = 14)	3	3	8	—	2	6 (43%)
Other causes (*n* = 2)	—	—	2	—	1	1 (50%)

*Note:* **Long‐term outcomes according to the factors leading to temporary stoma creation**. Abbreviations: APR, abdominoperineal resection; SC, stoma closure.

### 3.7 Stoma‐Free Survival

**FIGURE 2:**
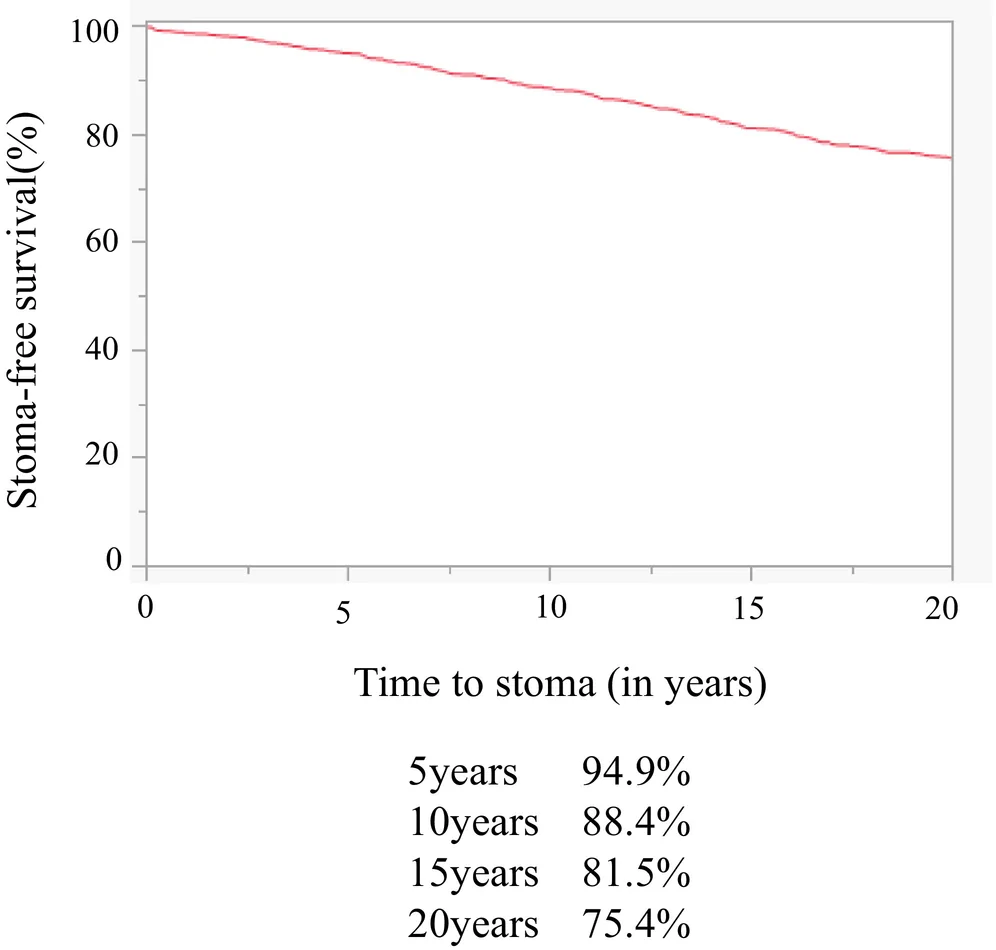
Stoma‐free survival from the time of Crohn's disease diagnosis. Stoma‐free survival was defined as the time to first stoma creation, including both temporary and permanent stomas.

Stoma‐free survival was defined as the time to first stoma creation, including both temporary and permanent stomas, and was evaluated from the time of Crohn's disease diagnosis. The stoma‐free survival rates were 94.9% at 5 years, 88.4% at 10 years, 81.5% at 15 years, and 75.4% at 20 years (Figure 2).

### 3.8 Permanent Stoma‐Free Survival

**FIGURE 3:**
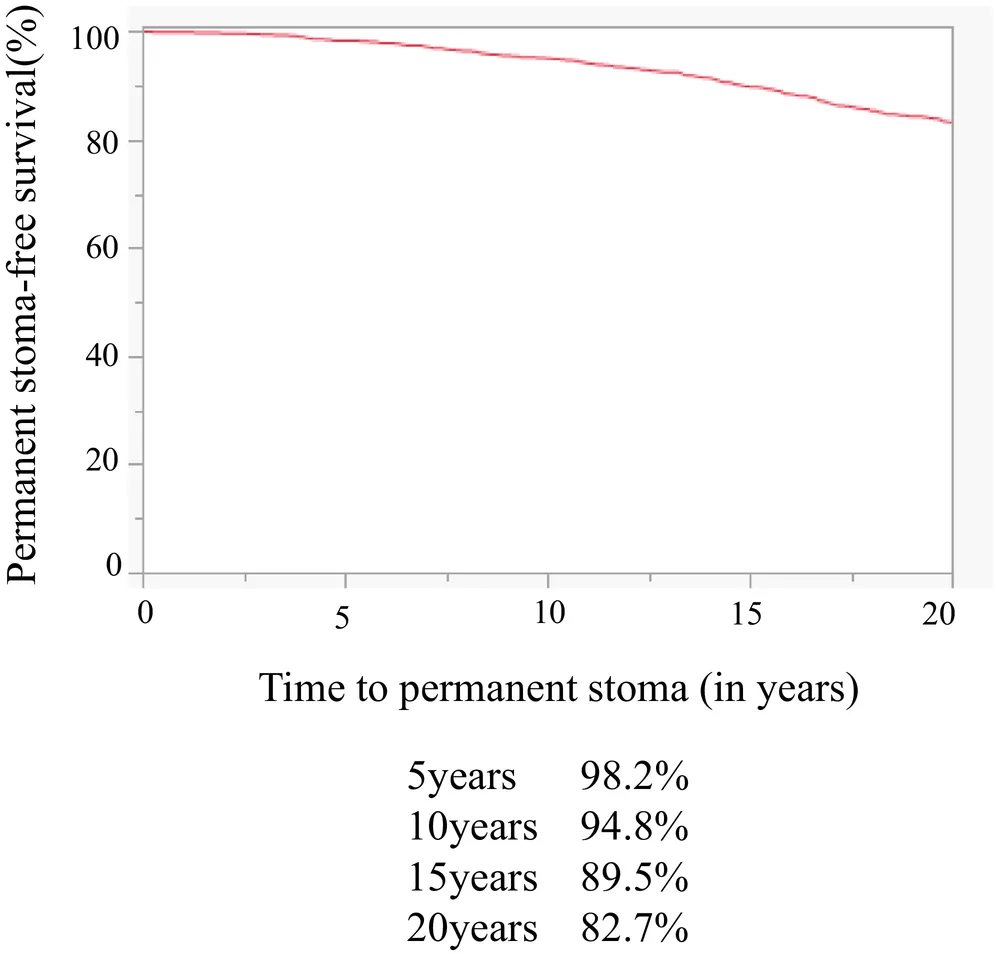
Permanent stoma‐free survival from the time of Crohn's disease diagnosis.

Among the 1440 patients who underwent intestinal resection for CD, the permanent stoma‐free survival rates were 98.2% at 5 years, 94.8% at 10 years, 89.5% at 15 years, and 82.7% at 20 years. The median follow‐up period for the cohort was 19.5 years (range, 7–50 years) (Figure 3).

**FIGURE 4:**
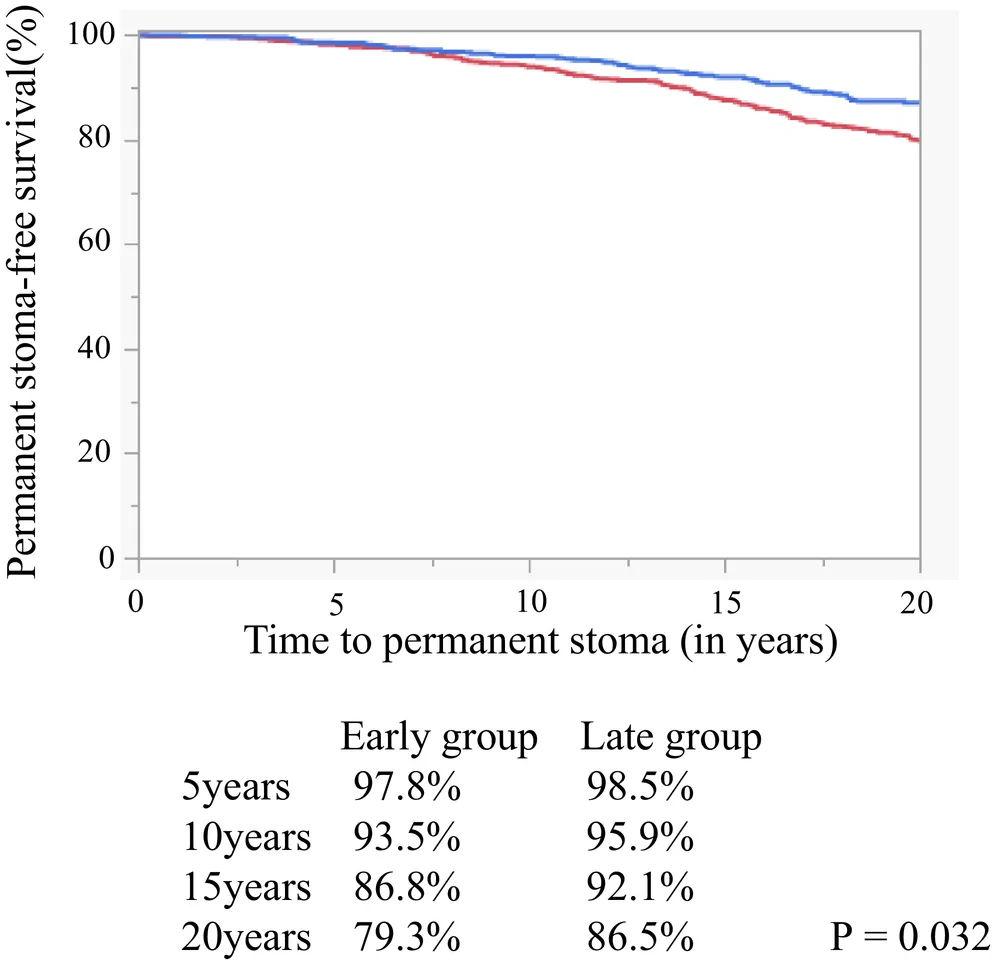
Permanent stoma‐free survival by surgical era from the time of Crohn's disease diagnosis.

When the patients were divided into two groups according to the biologic era, defined by the widespread clinical adoption of infliximab in Japan (before 2005 and after 2005), the 5‐, 10‐, 15‐, and 20‐year permanent stoma‐free survival rates in the early group were 97.8%, 93.5%, 86.8%, and 79.3%, respectively, whereas those in the late group were 98.5%, 95.9%, 92.1%, and 86.5%, respectively. Permanent stoma‐free survival was significantly greater in the late group than in the early group (*p* = 0.032) (Figure 4).

## 4 Discussion

In this study, we investigated the long‐term outcomes of stoma creation in patients with Crohn's disease (CD) using a large cohort of 422 patients with a median follow‐up of 19.5 years (range, 7–50 years). Among the 1440 patients who underwent intestinal resection, 422 (29.3%) required stoma creation during their disease course. The most frequent indication was the progression of anorectal disease, and 87% of the stomas created for this reason ultimately became permanent. The cumulative permanent stoma rates were 13% at 10 years and 27% at 20 years, which highlights the substantial long‐term surgical burden among CD patients with anorectal involvement. CD often presents at a young age and with diverse manifestations, such as strictures, fistulas, abscesses, and anorectal lesions. During the disease course, stoma creation is frequently required because of the progression of anorectal disease or impaired bowel function. In Asia, including Japan, anorectal lesions are particularly common, and the reported stoma creation rates range from 10.6%–37% in Japan and 20%–62% in Western countries [14, 15, 16]. In the present study, worsening anorectal disease accounted for more than half (53.1%) of all permanent stoma creations, thus reflecting both the high prevalence and clinical impact of anorectal involvement in Japanese patients. Previous studies from Japan have demonstrated that perianal disease is frequently diagnosed prior to the diagnosis of Crohn's disease. Mizushima et al. reported that a substantial proportion of Japanese patients present with perianal disease before intestinal manifestations become clinically apparent [17]. This characteristic clinical course may partly explain the high prevalence of anorectal disease and the subsequent need for stoma creation observed in our cohort.

We defined a permanent stoma as one that had not closed within 24 months after creation, which resulted in higher rates than those previously reported. This may be attributed to our focus on surgical cases and the high incidence of anorectal lesions among Japanese CD patients, as well as the long‐term cumulative effects of disease progression [15, 18]. The introduction of biologic therapy has markedly changed the treatment landscape of CD. Infliximab became available in Japan in 2002, thus leading to improved control of intestinal and anorectal inflammation [19]. Consistent with this, our study demonstrated a significantly lower cumulative permanent stoma rate in the biologics era, which suggests that advances in medical therapy may have contributed to better surgical outcomes. The introduction of biologic therapy has markedly changed the medical management of Crohn's disease; however, its impact on stoma‐related outcomes appears to differ depending on the disease stage and patient population. Population‐based data have shown that, despite increased use of biologics, the early risk of stoma creation after diagnosis has not substantially declined over time [9]. In contrast, our surgically treated cohort demonstrates that although biologic therapy may contribute to improved outcomes in selected patients, a substantial proportion—particularly those with anorectal disease—still progress to permanent stoma creation, highlighting the limited ability of current medical therapy to alter long‐term surgical outcomes in advanced disease. From a clinical perspective, effective control of anorectal disease requires a coordinated approach combining surgical and medical management. Following appropriate surgical interventions, such as seton placement for complex fistulas and local drainage for abscesses, inflammatory activity should be controlled using biologic agents, immunomodulators, and antibiotics. However, in advanced‐stage disease with irreversible anorectal damage, current therapeutic strategies alone may be insufficient to prevent permanent stoma creation. These findings highlight the importance of early and close collaboration between gastroenterologists and colorectal surgeons to optimize disease control and surgical decision‐making.

Overall, 31% of patients underwent stoma closure, but 13% required recreation. The stoma recreation rate was notably high (56%) among those who required an initial stoma because of anorectal disease, and 87% ultimately required a permanent stoma over the long‐term follow‐up period. Recent data on temporary fecal diversion for refractory anorectal Crohn's disease have also been reported. Kuroki et al. demonstrated that although symptom improvement was achieved in 74% of patients after fecal diversion, successful stoma closure was observed in only 2.2%, and more than 40% of patients ultimately required abdominoperineal resection [20]. These findings are consistent with our results, in which the majority of stomas created for anorectal disease eventually became permanent, underscoring the limited long‐term reversibility of fecal diversion in this patient population. In contrast, patients who underwent stoma creation for poor bowel condition or anastomotic leakage were more likely to maintain bowel continuity, and of these, 56% and 43% avoided permanent diversion, respectively. These findings emphasize the need for cautious decision‐making regarding stoma closure in patients with anorectal disease and highlight the importance of achieving optimal disease control before closure is attempted [21].

Cancer associated with CD has also emerged as an important clinical issue. In our cohort, 13% of patients had cancer related to anorectal disease. Surveillance for CD‐associated cancer remains challenging because strictures and pain often hinder endoscopic evaluation [22, 23]. Moreover, cancer can develop in diverted or excluded rectal segments following stoma creation [24, 25]. In our study, two such cases were identified, thus underscoring the necessity of continuous follow‐up for diverted rectal segments. While effective surveillance programs have been established for ulcerative colitis, standardized surveillance for CD‐associated cancer remains inadequate [26]. For patients with anorectal disease, close collaboration between gastroenterologists and colorectal surgeons is essential so that regular perianal examinations are incorporated, and, when necessary, evaluations under anesthesia are performed to facilitate early detection of malignancy.

This study has several limitations. First, it was a retrospective, single‐center analysis; therefore, selection and information biases cannot be completely excluded. Second, as our institution is a tertiary referral center that specializes in inflammatory bowel disease, the cohort may include a greater proportion of severe or refractory cases, which limits generalizability. Third, changes in medical and surgical management over the 40‐year study period may have influenced the outcomes. In addition, the exact onset of anorectal disease could not be precisely determined in all patients because of the retrospective nature of the study and incomplete documentation of perianal disease history, particularly in patients referred from other institutions; therefore, the duration of anorectal disease was not separately analyzed. Nevertheless, the strengths of this study lie in its long‐term follow‐up period and large sample size, which allowed us to clarify the real‐world clinical course and prognosis of stoma creation in patients with CD. Our findings reinforce the critical importance of achieving effective control of anorectal disease activity. Given the continued advancement of multidisciplinary approaches, including biologic therapy, the further reduction in the need for stoma creation and permanent diversion in patients with Crohn's disease may become possible.

## 5 Conclusion

In this study of 422 patients with Crohn's disease, we investigated the long‐term outcomes of stoma creation. The most frequent indication was the progression of anorectal disease, and 87% of these stomas ultimately became permanent. Appropriate control of anorectal disease may help prevent stoma creation and permanence, thereby improving the long‐term prognosis of patients with Crohn's disease.

## Author Contributions

Conception and design: K. Nagano, M. Uchino, R. Kuwahara, H. Ikeuchi. Data acquisition: K. Nagano, R. Kuwahara, Y. Horio, K. Kusunoki, Y. Tomoo. Data analysis and interpretation: K. Nagano, R. Kuwahara, M. Uchino. Drafting the article: K. Nagano, R. Kuwahara, H. Ikeuchi. Critical revision of the article: K. Nagano, R. Kuwahara, M. Uchino, H. Ikeuchi, Y. Horio, K. Kusunoki, Y. Tomoo. Final approval of the article: K. Nagano, R. Kuwahara, M. Uchino, H. Ikeuchi, Y. Horio, K. Kusunoki, Y. Tomoo. All figures and tables are original and were generated by the authors.

## Funding

The authors have nothing to report.

## Ethics Statement

All study protocols were approved by the Institutional Review Board of Hyogo Medical University (No. 4678).

## Consent

This study featured a retrospective design, and thus the requirement for consent for participation was waived; however, patients were given the opportunity to opt out.

## Conflicts of Interest

The authors declare no conflicts of interest.

## Data Availability

The data that support the findings of this study are available on request from the corresponding author. The data are not publicly available due to privacy or ethical restrictions.
